# A prospective study integrating a curriculum of interventional radiology in undergraduate education: a tetra-core simulation model

**DOI:** 10.1186/s42155-020-0104-y

**Published:** 2020-03-09

**Authors:** Iakovos Theodoulou, Christina Louca, Michail Sideris, Marios Nicolaides, Deepsha Agrawal, Antonios Halapas, Athanasios Diamantopoulos, Apostolos Papalois

**Affiliations:** 1 Faculty of Life Sciences and Medicine, King’s College London, Great Maze Pond, London, SE1 9RT UK; 2 Department of Interventional Radiology, Guy’s and St Thomas’ Hospitals, NHS Foundation Trust, London, UK; 3 Barts and the London School of Medicine and Dentistry, Queen Mary University of London, London, UK; 4 County Durham and Darlington NHS Foundation Trust, Darlington, UK; 5 Department of Transcatheter Heart Valves, Hygeia Hospital, Athens, Greece; 6 Experimental Educational and Research Centre ELPEN, Athens, Greece; 7 School of Medicine, European University Cyprus, Nicosia, Cyprus

**Keywords:** Simulation, Undergraduate, Curriculum, Interventional radiology, Procedural skills

## Abstract

**Background:**

Interventional radiology (IR) is underrepresented in undergraduate medical curricula across Europe. By continuing to challenge the boundaries of IR, a rise in the demand for radiologists has been inevitable – a trend not met by a corresponding rise in the supply of radiologists. On tracing the roots of this shortage, lack of awareness of the specialty within medical trainees coupled with a global lack of IR teaching in undergraduate education seem to constitute major exacerbating factors. The purpose of this study was to identify gaps in the field of IR education and address these by implementing an international IR simulation-based course for undergraduates.

**Results:**

Implementation of a multi-modality simulation-based course consisted of seven modules incorporating technical and non-technical skills, basic science and applied clinical science modules. Of all participants, 90.7% (*N* = 68) never had previous IR teaching experience and only 28% (*N* = 21) had a previous placement in an IR department. Following the course, confidence improvement was statistically significant both in IR skills (1/5, *p* < 0.01) and knowledge (1/5, *p* < 0.01)]. The majority (90.7%) said they would benefit with more exposure to IR. In terms of the students’ motivation for a career in IR, 32% (*N* = 24) reported that they would more likely consider a career in IR after completing the course.

**Conclusion:**

Delivery of a tetra-core simulation course with the aim to address the gaps in undergraduate IR education has had a positive impact on students’ skills, confidence levels as well as motivation. We propose reviewing the curricula across medical schools in Europe to identify gaps and address any inadequacies; for this, we consider our simulation course an excellent starting point.

## Background

Interventional radiology (IR), officially granted sub-specialty status since 2010, is a field with a rapidly expanding potential, and an ever-increasing significance in modern healthcare delivery (Leong et al. 2009; Lee and Lee 2018). By continuing to challenge the boundaries of IR, a rise in the demand for radiologists has been inevitable – a trend not met by a corresponding rise in the supply of radiologists. For instance, in 2018 there was a 23% shortfall of radiologists, which is only estimated to rise to 31% by 2023 (The Royal College of Radiologists 2018). Furthermore, a recent report by the Royal College of Radiologists (RCR) in the United Kingdom (UK) analyses the negative impact such shortages may have both on patient care and hospital services. Indeed, it is expected that prolonged staffing shortages will simply prevent hospitals from accessing IR services within or across sites (The Royal College of Radiologists 2018).

On tracing the roots of this shortage, lack of awareness of the specialty within medical trainees coupled with a global lack of IR teaching in undergraduate education seem to constitute major exacerbating factors (Muzumdar et al. 2019). For example, in the UK there is currently no defined undergraduate IR curriculum, whilst the European Society of Radiology (ESR) found that an average of just 5.3 hours are dedicated to radiology at the undergraduate level. Similar worrying facts are echoed in Atiiga et al. and Leong et al. who found that only 43.2 and 35% of medical students respectively, had an experience with IR (Leong et al. 2009; Atiiga et al. 2017). Similar gaps appear in medical students’ knowledge on common IR procedures with knowledge failing to meet the 50% pass mark (Alsafi et al. 2017). These findings highlight the need for introducing a standardized undergraduate curriculum to bridge the knowledge and skills gap between IR and other specialties as well as to eliminate any inconsistencies in IR teaching across medical schools in Europe (Alsafi et al. 2017; Kourdioukova et al. 2011).

Similarly to surgery, IR training via simulation, allows for skills to be practiced and evaluated in a controlled environment by identifying strengths and weaknesses. Previous reports suggest that incorporating IR simulation in undergraduates greatly enhances the skill-set of students planning to follow a career in IR as well as those pursuing allied interventional specialties such as cardiology, anaesthetics and intensive care (Maingard et al. 2017). In fact, up to 19% of newly qualified juniors end up in interventional specialities (The Royal College of Radiologists 2018). Coupled with the wide applicability of IR skills and principles, there is now a strong case to counter previously voiced concerns about IR training constituting a ‘step too far’ for the undergraduate student.

The abovementioned ‘troubled’ landscape forms the basic driver of the rising efforts for improved radiology teaching in medical school as well as the reshuffling of radiology postgraduate training pathways (Muzumdar et al. 2019; Alsafi et al. 2017). Relevantly, the Cardiovascular and Interventional Radiological Society of Europe (CIRSE) has published the first undergraduate IR curriculum to guide the integration of IR into undergraduate teaching (CIRSE 2018); however, there remains scarcity of data around its implementation. Inspired by CIRSE’s recent report, the aims of this study were firstly, to ascertain students’ existing knowledge and understanding of IR; secondly, to examine overall motivation to follow a career in IR; and lastly, to determine the extent to which our proposed course curriculum improves students’ knowledge, skills and reported confidence levels in a pre-defined skillset.

## Methods

### Pilot

The scarcity of data regarding IR undergraduate curricula necessitated a preliminary qualitative pilot study to ascertain the current landscape. Through a systematic literature review (Emin et al. 2019), followed by structured interviews and student questionnaires we were able to identify gaps in the field. Seventy-nine students and five faculty members participated in the pilot over a period of 8 months. The aim of the pilot project was twofold: firstly, to ascertain the current landscape in undergraduate education in IR by exploring students’ and doctors’ views about the possible launch of such a course and secondly, to actually launch a ‘test’ course which would be smaller in scale (less skills stations) and shorter in duration. This would reveal ideas, flaws and things to work upon for the true course. Following this, a curriculum which would form the backbone of the study was discussed and finalised by the course faculty comprising of consultants in Interventional Radiology and Cardiology.

### Study setting

The current course setup was inspired from and designed around the principles of ESMSC - a four-day surgical simulation course for undergraduate students. The course concept and student selection in ESMSC has been described elsewhere (Sideris et al. 2018). For the purpose of our study, a total of 75 undergraduate medical students participated of whom 61.3 and 38.7% were females and males respectively. IRB approval was sought and granted after the application met directive 63/2010, PD 56/April 2013 declaration. While recruitment of participants was multi-site, the actual course took place in a single simulation centre, bringing students together from all around Europe. Therefore, given that the facilities of the centre were unchanged, consistency across simulation modules was ensured.

### Tetra-core curriculum

**Fig. 1:**
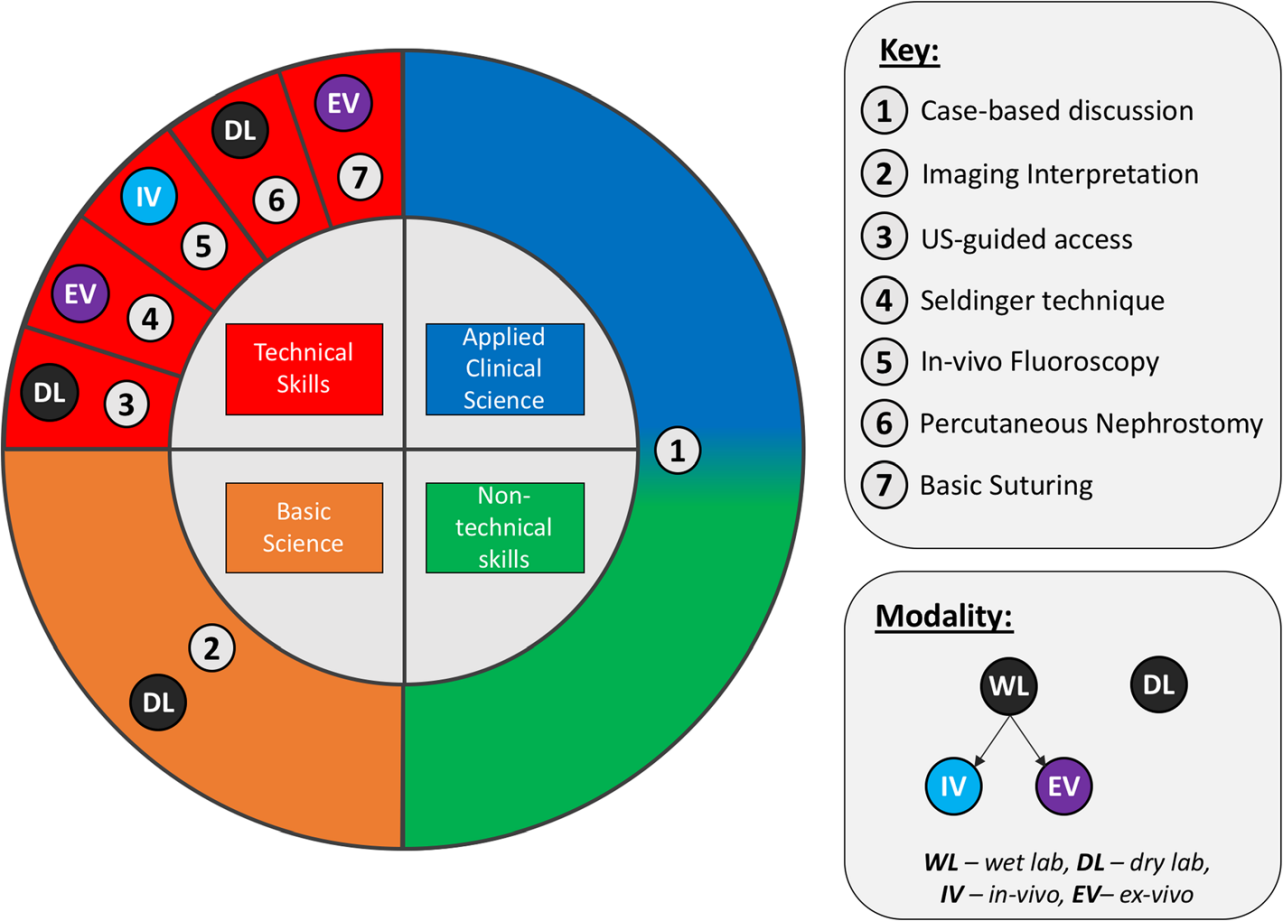
Course curriculum. Course curriculum mapped onto the four cores of Ci4R with inner quadrants indicating the four cores of the framework and outer coloured sections indicating the different stations

**Fig. 2:**
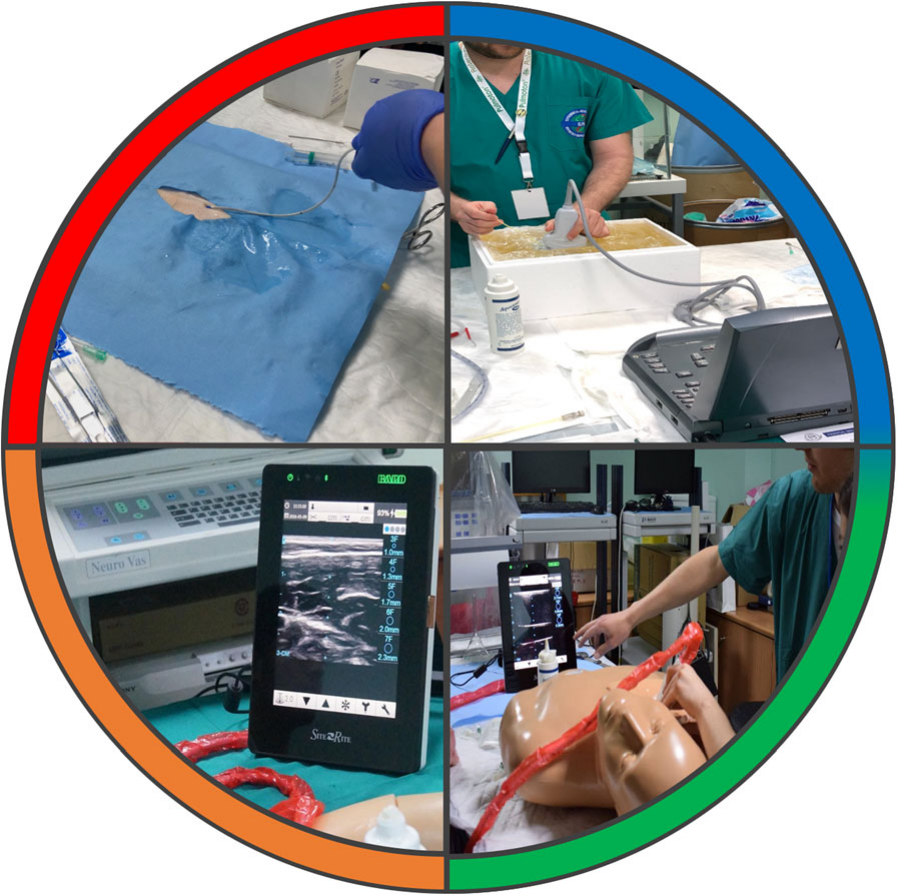
Modules employed in each of the four cores of the curriculum. A pictorial representation of some of the different modules employed in each of the four cores of the curriculum

This multi-modality simulation course of IR in undergraduates was conceived and launched in April 2019, the structure of which is illustrated in Figs. 1 and 2 and Table 1 . Inspired by the Ci4R framework (Sideris et al. 2018), this novel course consists of seven teaching modules incorporating skills from all four cores of Ci4R: technical skills, non-technical skills, basic science and applied clinical science. Technicalities surrounding the precise setup of this course are beyond the scope of this article and are best described by Sideris et al. (Sideris et al. 2018).

**Table 1:** Summary of our IR simulation course curriculum

Module	Core	Duration	Coordinators
Team dynamics & the IR team	Non-technical Skills	30′	1
Catheters and sizing in IR	Technical Skills	30′	2
Coils, plugs and embolization materials	Technical Skills	30′	2
Revision of Vascular Anatomy	Basic Science	30′	1
Suturing Station	Technical Skills	30′	2
US Guided Venous Access	Technical Skills	30’	2
Seldinger Technique	Technical Skills	30’	2
In-vivo Cath Lab Station	Technical Skills	30’	3
Nephrostomy Station	Technical Skills	30’	3
X-ray Interpretation	Applied Clinical Science	30’	1
Fluoroscopy Image Interpretation	Applied Clinical Science	30’	1
IR cases and troubleshooting	Case-based Discussion	30’	1

### Ethical approval

All procedures involving human participants were in accordance with the ethical standards of the 1964 Helsinki declaration. All applicable international, national, and/or institutional guidelines for the care and use of animals were followed. The ESMSC course is compatible with the current 3R principles for animal-model simulation (refinement, replacement and reduction). Application of ethical approval met directive 63/2010, PD 56/April 2013 declaration, according to local policy. The license reference number is 4857/15-09-2017, MS, AP et al.

### Data collection

Anonymous questionnaires were distributed electronically to all participants prior to the course. These were designed to elicit students’ awareness and existing knowledge of IR as well as self-reported confidence levels on skills which were to be assessed during the course, as listed in Fig. 1. During the course, all students underwent objective assessment by two qualified Radiologists, using the Direct Observation of Procedural Skills (DOPS) - a validated tool of workplace-based assessment in the UK. DOPS were performed before and after a simple intervention: demonstration of the skill by the attending Radiologist, a short tutorial and the opportunity to practise for 5 min. Data collection was concluded with post-course questionnaires eliciting students’ views on the overall experience and perceived improvements in their knowledge, skills and motivation for IR.

### Statistical analysis

Univariate inferential statistics were used to describe data; we used non-parametric associations as sample size and normality test indicated so (Sapiro-Wilk test). Numerical data were associated with Spearman’s rho, and further exploration of median values was performed using Wilcoxon (paired associations) or Mann-Whitney (2-independent groups) or Kruskal Walis (*k-*independent groups). All data were analysed using IBM SPSS for Macintosh v.25.0 (IBM corp., Armonk, NY, USA).

## Results

### Demographics & previous exposure

Of the total sample, 88% (*N* = 66) of students were in clinical years while the remaining 12% (*N* = 9) were in their preclinical years. All students attended European Medical Schools with 41.3% (*N* = 31) in the UK, 42.7% (*N* = 32) in Greece, and the remaining in other European countries. A noteworthy association was the relative superiority in Seldinger DOPS in students currently in the pre-clinical years (*p* = 0.049). Further exploration of this association with a Mann-Whitney reduced this finding to only marginally significant (*p* = 0.051).

In terms of previous exposure, 90.7% (*N* = 68) never had previous IR teaching experience even though 72% (*N* = 54) agreed that IR requires a strong set of technical skills. Similarly, only 28% (*N* = 21) had a previous placement in an IR department – a stark contrast to the 100% who attended surgical lists. This was reflected in the marked heterogeneity in students’ answers for the definition of IR– for example, 37.3% (*N* = 28) described IR as a ‘medical specialty’. On asking students to classify different procedures as either ‘surgical’ or ‘radiological’ the heterogeneity of factual accuracy was remarkable. Overall, noted pre-course variables were not found to impact performance in any way.

### Awareness, confidence and skills

Following teaching, confidence improvement was statistically significant both in IR skills and knowledge with a median improvement of 1/5 (IQR: 1, *p* < 0.01) and 1/5 (IQR: 2, *p* < 0.01) respectively (Table 2). Equally, improvement was statistically significant across all three skills with median improvements of 2/10 (IQR = 2; *p* < 0.01), 6/10 (IQR = 2; *p* < 0.01) and 7/10 (IQR = 2; *p* < 0.01) in USS Access, Seldinger and Nephrostomy stations respectively.

**Table 2:** Correlations between pre-course variables and total DOPS Scores

	Total DOPS Score improvement in:					
	USS Access		Seldinger		Nephrostomy	
	Rho	*p*-value	Rho	*p*-value	Rho	*p*-value
Demographics						
Male/Female	−0.068	0.560	0.168	0.319	0.025	0.883
Year of studies	0.219	0.059	−0.325	0.049	−0.121	0.474
Country	−0.097	0.409	0.066	0.699	−0.117	0.491
Age	−0.159	0.172	−0.047	0.782	−0.012	0.942
Exposure						
Previous teaching	0.217	0.062	−0.194	0.250	0.019	0.911
Previous attachment	0.035	0.764	0.029	0.863	0.282	0.091
Confidence						
US Access Skills	−0.170	0.145	n/a	n/a	n/a	n/a
Knowledge	0.079	0.501	−0.255	0.127	0.172	0.309
Knowledge						
Factual accuracy	−0.044	0.708	0.058	0.733	0.175	0.300
Motivation						
Likelihood to consider IR	0.124	0.291	−0.133	0.434	−0.035	0.835

**Table 3:** Associations between improved confidence and DOPS improvement

	Total DOPS Score improvement in:					
	USS Access		Seldinger		Nephrostomy	
	Median = 2; IQR = 2; *p* < 0.01		Median = 6; IQR = 2; *p* < 0.01		Median = 7; IQR = 2; *p* < 0.01	
	coefficient	*p*-value	coefficient	*p*-value	coefficient	*p*-value
Skills Confidence improvement	0.166	0.155	−0.161	0.343	0.046	0.787
Median = 1; IQR = 1; *p* < 0.01						
Knowledge Confidence improvement	0.024	0.839	−0.056	0.740	−0.036	0.834
Median = 1; IQR = 2; *p* < 0.01						

In capturing students’ self-reported confidence for a predefined skillset, we were also able to compare this with their actual DOPS performance and assess for associations. The only statistically significant association observed was between lower confidence levels on IR knowledge and overall performance improvement in the Seldinger station (*p* = 0.049). However, on further exploration using a Kruskal-Wallis test, this finding was dismissed as non-significant (*p* = 0.695).

Furthermore, skills considered to be essential for effective demonstration of US-guided access, such as appropriate probe selection, probe handling and good Seldinger technique, were all self-rated very low prior to entering the station. For example, self-reported skill level and knowledge of US had a median of 2/5 (IQR =1) and 2/5 (IQR = 1,), respectively. Whilst self-reported confidence levels showed a significant improvement upon practical exposure of students in the relevant stations, no statistically significant correlations could be identified with the positive trends in DOPS performances.

### Motivation for a career in IR

The third part of data collection aimed to assess the potential of this educational initiative to stimulate students’ interest in a career in IR. An impressive 68% (*N* = 51) of students never considered IR as a career option, but more striking was the reason for this: 22.7% (*N* = 17) admitted that no one ever encouraged them to think about this possibility. Other important findings included an overwhelmingly positive response (90.7%) when asked whether they would benefit with more exposure to IR during medical school teaching. In a similar way, 77.3% (*N* = 58) of students reported they would support the implementation of a mandatory module or placement in IR. Lastly, in assessing the extent of the impact the course had on students’ perception of IR, 32% (*N* = 24) reported that they would more likely consider a career in IR, however, the increased likelihood was not found to be correlated with evident DOPS improvements.

## Discussion

The need to address IR in undergraduate education has been a topic of debate for some time with various studies reporting a profound lack of IR teaching or worrying levels of knowledge amongst students (Atiiga et al. 2017; Alsafi et al. 2017). These and similar findings eventually led to the publication of ‘*IR Curriculum for Medical Students’* in 2012 (Shaikh et al. 2016). Despite initial enthusiasm, a systematic review in 2019 concluded that 7 years later, the report has failed to make a substantial impact, with IR curricula remaining largely untouched (Emin et al. 2019).

The structure and delivery of undergraduate curriculum are critical determinants of the type of medical graduates we produce. Amongst the extensive skills and knowledge doctors are expected to carry to their first jobs, one attribute that remains neglected is *clinical acumen*. This can be defined as the ability to navigate through clinical decision-making, utilizing skills and knowledge while remaining cognizant of the roles of different clinical specialties. We postulate that clinical acumen is the product of holistic undergraduate training, that is, enabling students to think beyond textbooks and exposing them to the realities of clinical practice. The absence of such elements subsequently leads to graduates with an underdeveloped clinical acumen.

As shown in Fig. 1, the cyclical course design lent itself for introducing stations with increasing complexity in a step-wise fashion. A unique aspect of our course was the implementation of an in-vivo porcine simulation module. With the exception of the in-vivo module, many of the simulation stations were inspired from previous studies (Rock et al. 2010; Sideris et al. 2015). This was perceived by students as ‘inspiring’, offering the ‘best possible insight into IR’. While fluoroscopy is a skill that no junior doctor will be expected to undertake, this module succeeded in its purpose by instigating a thought process in students’ minds about the capabilities of IR and its place in clinical practice. Relevantly, 68 % of students did not consider IR as a potential career prior to the course, the main reason for this being a mere absence of opportunities to see the specialty in practice. If in-vivo simulation can make up for this lack of opportunities, does it not deserve a place in undergraduate curricula? Nevertheless, we do recognize both the cost and ethical implications educators and policy-makers may encounter when trying to adopt such practices.

The study employed a comprehensive set of outcomes before and after the course, in order to quantify students’ clinical acumen in relation to IR. With the goal of this course being to improve students’ attributes across all four outcomes, the positive results have reassured us of its effectiveness. Although no correlations were identified between pre-course variables and DOPS scores (Table 2), we postulate that in order for potential associations to be revealed, a formal, larger scale curriculum may be needed.

Moreover, the plurality of the metrics not only helped capture a spherical view of the current landscape in undergraduate IR training, it also enabled us to examine intricate relationships between different metrics such that those which merit greater attention are prioritised in future efforts. For example, confidence improvement was not associated with performance improvement in any way – a finding which again pointed to the limited size of our sample. This signifies the need for larger studies and dissemination of the course to validate potential associations.

Associations between confidence improvement and DOPS scores (Table 3), albeit non-significant, did show a positive correlation, which begs the question: would a bigger sample alter the significance levels? If so, confidence levels can be assumed to positively impact on student performance. As seen in previous studies, positive performance can influence motivation in certain specialties (Patel et al. 2013; Drolet et al. 2014; Day et al. 2016). In other words, one should not regard the aim of this course as solely performance-boosting. Rather, this initiative should be regarded as confidence-enhancing, offering students opportunities to explore previously unexplored areas. Be it in the form of confidence improvement, or indeed performance improvement, such metrics may encourage students to consider new careers like IR, or at least reconsider what we suggest could be ‘immature’ career choices. These ideas are echoed in earlier studies, where early introduction of IR lectures has increased both awareness and interest in the specialty (Shaikh et al. 2016; Branstetter IV et al. 2007).

Furthermore, with the majority never having completed an attachment to IR, our study found that there is a massive lack of knowledge about IR’s role in clinical practice. As supported by previous studies, ignorance about the specialty ultimately leads to a lack of interest in it altogether (Alsafi et al. 2017; Emin et al. 2019). Therefore, implementation of a carefully revised curriculum which enables individuals to experience the practical side of IR, can be catalytic in demystifying the specialty and increasing recruitment. In fact, more than half of the participants supported the idea of introducing more IR modules or placements. However, being a multi-centre study, the study sample does carry an element of self-selection and potentially some polarisation of opinions.

Moving forwards, there are two important questions to address; the first one being the very ways in which we go about introducing such courses in existing medical school curricula. This adaptation can take various forms, either through more isolated ‘simulation days’ or more systematic approaches, supplementing the latter with placements in IR departments. IR deserves dedicated placements of, perhaps, shorter duration compared to core medical specialties, but still of enough duration to raise students’ awareness of what IR entails. This, in turn, requires rethinking of what seems like an ‘overcrowded’ curriculum, going as far as challenging the current duration of placements like Vascular surgery, and recognising the fact that as IR expands, such areas are constricting. The second challenge concerns the lack of recognition of IR as an official specialty, at least in the UK. This forms the main hinge of hinderance for all efforts to ‘make the case’ for more IR teaching, which is why recent talks for segregation of IR as a standalone specialty are so crucial. Specialty status will expedite the creation of learning outcomes for undergraduates specific to IR and embrace courses like the current study.

## Conclusion

Through the formal implementation of our tetra-core simulation course, our study is one of the first to embrace CIRSE’s latest recommendations in an attempt to address current gaps in undergraduate IR education. Importantly, the incorporation of simulation elements in our curriculum has been two-fold: not only did it form a unique and unprecedented platform for the acquisition of essential practical skills, it also enabled students to explore the nature of the specialty, build up confidence and appreciate previously unfamiliar concepts. Encouraged by the positive impact of our course, we propose that curricula across Europe are reviewed such that gaps are identified and changes are introduced to address any inadequacies; for this purpose, we consider our concept map as an excellent starting point.

## Data Availability

Data can be made available upon request.

## Abbreviations

CIRSE: Cardiovascular and Interventional Radiological Society of Europe
DOPS: Direct Observation of Procedural Skills
ESMSC: Essential Skills in Management of Surgical Cases
ESR: European Society of Radiology
IR: Interventional Radiology
IRB: Institutional Review Board
RCR: Royal College of Radiologists
